# Primary Hyperparathyroidism and Hyperthyroidism in a Patient with Myotonic Dystrophy: A Case Report and Review of the Literature

**DOI:** 10.1155/2015/735868

**Published:** 2015-06-15

**Authors:** Yosra Cherif, Baha Zantour, Wafa Alaya, Olfa Berriche, Samia Younes, Mohamed Habib Sfar

**Affiliations:** Department of Endocrinology and Internal Medicine, Tahar Sfar University Hospital of Mahdia, Hiboun District, 5100 Mahdia, Tunisia

## Abstract

Various endocrine manifestations are commonly described in myotonic dystrophy (MD), including primary hypogonadism, diabetes mellitus, and thyroid and parathyroid dysfunction. We describe a 46-year-old woman with a family history of MD with her son. She was diagnosed with cardiac arrhythmia and required the implantation of a pacemaker. She was noted to have a bilateral cataract. She complained of muscle weakness, diffuse myalgia, and palpitation. The electromyography (EMG) showed myotonic discharges. Laboratory tests showed high serum calcium 2.83 mmol/L, serum phosphate 1.2 mmol/L, parathormone 362.5 pg/mL, thyroid stimulating hormone TSH 0.02 mIU/L (normal range: 0.34–5.6 mIU/L), FT4 21.17 ng/mL, and negative anti-thyroperoxidase antibodies. Cervical ultrasound revealed a multinodular goiter. The 99mTc-MIBI scintigraphy localized a lower right parathyroid adenoma. The clinical data, the family history of MD, EMG data, and endocrine disturbances were strongly suggestive of MD associated with hyperthyroidism and primary hyperparathyroidism.

## 1. Introduction

Myotonic dystrophy (MD) is an autosomal dominant disorder that results from an expanded CTG repeat in a myotonic dystrophy protein kinase (DMPK) gene on chromosome 3 or 19. It is the most common muscular dystrophy in adults. The disease is characterized by muscle weakness, dystrophic changes in neuromuscular tissues, frontal baldness, cataracts, cardiac disorder, and mental impairment with the development of the disease process. Various endocrine manifestations are commonly described, including primary hypogonadism, diabetes mellitus, and thyroid and parathyroid dysfunction [1]. A few cases of hyperthyroidism or hypothyroidism associated with MD have been reported, but there are only 2 reports, to our knowledge, concerning MD associated with primary hyperparathyroidism (PHP) and hyperthyroidism [2, 3]. Herein, we describe an association of PHP and hyperthyroidism in a patient with MD and we reviewed all cases reported in the literature.

## 2. Case Report

A 46-year-old woman was admitted in 2010 for investigation of hypercalcemia discovered during recurrent nephrolithiasis. Her family history was noteworthy with cardiac arrhythmia in two dead sisters and nephrolithiasis in another sister. Her son was diagnosed with MD at the age of 24 years. Five years ago, an arrhythmia was diagnosed and required the implantation of a pacemaker. She was noted to have a bilateral cataract. She complained of muscle weakness, diffuse myalgia, and palpitation. The weakness was gradually progressive in arms. Physical examination revealed percussion myotonia and the typical emaciated face of MD, hollow cheeks, drooping jaw, a peripheral neurogenic syndrome, and multinodular goiter. There was no exophthalmia. The electrocardiogram was normal. Hypercalcaemia was confirmed 2.83 mmol/L. There were no symptoms directly attributable to hypercalcemia. Other laboratory tests showed serum phosphate 1.2 mmol/L, urinary calcium 0.153 mmol/kg/day, creatinine level 52 *μ*mol/L, alkaline phosphatase 97 IU/L (normal range: 45–245 IU/L), parathormone 362.5 pg/mL (normal range: 15–65 pg/mL), thyroid stimulating hormone TSH 0.02 mIU/L (normal range: 0.34–5.6 mIU/L), FT4 21.17 ng/mL (normal range: 6.09–12.2 ng/mL), and negative anti-thyroperoxidase antibodies. Cervical ultrasound revealed a multinodular goiter with isoechogenic homogenic nodules with clear border and cystic cavities (8*∗*6 mm). The 99mTc-MIBI scintigraphy localized a lower right parathyroid adenoma. The electromyography (EMG) showed myotonic discharges. The bone mineral density (BMD) was normal. The clinical data, the family history of MD and arrhythmia in 2 sisters, EMG data, and endocrine disturbances were strongly suggestive of MD associated with hyperthyroidism and primary hyperparathyroidism (PHP). The patient was treated with benzylthiouracil, propranolol, and vitamin E. Euthyroidism was obtained after 2 years and the antithyroid drug was stopped. Four years later, MD is still in remission, and the alkaline phosphatase levels, serum creatinine and calcium, urinary calcium, and BMD are normal during follow-up.

## 3. Discussion

Our patient showed history, clinical, neurological, and electromyographic evidence of MD. Screening for endocrine disorders revealed PHP and hyperthyroidism.

The association of MD and endocrine and metabolic disturbances has been described by many authors [1]. Disorders of glucose metabolism were common and dominated by carbohydrates intolerance with hyperinsulinemia [1]; type 2 diabetes was less common (3%) [1]. Even so, thyroid dysfunction has been reported; we reviewed 56 cases (Table 1). Plasma FT4 was blunted in 12 patients with MD (Table 1). Isolated hyperthyroidism was reported in 7 cases and it was associated with Addison disease in 2 cases (Table 1). Ørngreen et al. studied the thyroid profile of 97 patients with MD and showed only 5 cases of hypothyroidism, 2 cases of hyperthyroidism, and 2 other cases of nontoxic multinodular goiter [1]. Single or multiple thyroid nodules with euthyroid status (33 patients) were found in several studies [4–7].

The parathyroid dysfunction is less common in patients with MD (Table 2). Cases of PHP have been reported in 16.5 and 18%, mainly parathyroid adenomas [1, 27]. Other few cases were reported [2, 28]. We recorded 34 cases of isolated PHP and 13 of pseudohypothyroidism in the literature (Table 2). Most of those patients exhibit parathyroid adenoma, and only one patient had parathyroid hyperplasia (Table 2).

The coexisting of several endocrinopathies with MD is rare (Tables 1 and 2). Association of Addison's disease and PHP was reported in 2 cases [8] (Table 1). In a review of the literature, we found one case of MD associated with hyperthyroidism and hyperparathyroidism (Table 1) and 2 cases of MD associated with PHP and thyroid carcinoma (Table 2). The first case described a patient who also suffered from hypergonadotropic hypogonadism and hyperinsulinism [2]. At surgery, a parathyroid adenoma was extirpated, and a subtotal thyroidectomy was performed [2]. Rosenberg et al. reported 2 cases of MD associated with parathyroid adenoma and thyroid carcinoma treated with parathyroidectomy and thyroidectomy [3]. To our knowledge, this publication is the third case report of MD associated with simultaneous hyperthyroidism and hyperparathyroidism.

Muscular disorders are often frequent in patients with thyrotoxicosis [34]. It can manifest as myotonic features [9, 34]. Several authors reported yet that the treatment of patients with hyperthyroidism improved myotonic symptoms [2, 9–11, 34]. Then, it may be hard to distinguish them from myotonia related to MD in the absence of electromyographic study. These myotonic discharges are nonspecific and can be experienced in other various diseases such as Duchenne muscular dystrophy, hypokalemic periodic paralysis, drug induced myotonia, and thyrotoxicosis [34–36]. Our patient had a family history of MD, cataracts, and cardiac disorders and she continued to show clinical evidence of myotonia 2 years after successful medical treatment with benzylthiouracil.

Steinbeck and Fukazawa suggested that MD may result from a defect in cellular membrane function [4, 5]. It is possible that the response to activation of TRH receptors on cell membrane is abnormal, which might explain the reduced TSH response to TRH and the increased incidence of goiter in even euthyroid patients with MD [4, 5]. Only one case report showed a larger amplification of CTG triplets in thyroid in a patient with MD and associated nodular goiter and suggests that repeated amplification of CTG triplet in thyroid tissue contributes to its dysfunction [12]. Ørngreen et al. showed a correlation between CTG expansion size with plasma PTH, phosphate, and serum calcium [1, 29]. Similar findings were described by Kinoshita et al. [37]. In addition, muscle weakness is a well-known complication of hyperparathyroidism. Passeri et al. showed a negative correlation between muscle strength in MD and PTH levels [27].

In conclusion, coexistence of hyperthyroidism and primary hyperparathyroidism may be more prevalent than what was previously recognized. Although further studies are needed to clarify the link between these two disorders and MD, the present case emphasizes the prominence of screening of parathyroid and thyroid function in patients with MD.

## Figures and Tables

**Table 1:** Thyroid disorder associated with MD: review of the literature.

Authors	Number of cases	Age/sex	Hormonal status	Clinical features	TSH *μ*U/mL	T4/T3	Treatment
Ørngreen et al. 2012 [1]	97	—	5: hypothyroidism 2: hyperthyroidism 2: nontoxic multinodular goiter	1: excessive tiredness	Low TSH: 5 (2%) High TSH: 2 (5%)	Normal/normal Normal/low	L-Thyroxine

Molina et al. 1996 [2]	1	—	Hyperthyroidism, hyperparathyroidism, hypergonadotropic hypogonadism, and hyperinsulinism	—	—	—	Parathyroid adenoma extirpation, subtotal thyroidectomy

Steinbeck and Carter 1982 [4]	20	38.3/—	2: nontoxic multinodular goiter 1: single thyroid nodule	Euthyroid	Euthyroid: 2.6 ± 0.5	101.5 ± 28/1.86 ± 0.57 nmol/L	—

Fukazawa et al. 1990 [5]	12	—	1: nontoxic multinodular goiter	Euthyroid	Euthyroid: 2.7 ± 1.3	16.6 ± 4.5/1.61 ± 0.29 nmol/L	—

Bonanni et al. 1997 [6]	33	—	9: nontoxic multinodular goiter 9: single thyroid nodule	Euthyroid	Normal	Normal/normal	—

Lee and Hughes 1964 [7]	19	—	4: nontoxic multinodular goiter 2: single thyroid nodule	—	—	—	—

Zargar et al. 2002 [8]	1	27/M	Hypothyroidism and Addison's disease	Euthyroid	—	—	—

Peterson et al. 1976 [9]	2	—	Hyperthyroidism	Thyrotoxicosis	—	—	—

Okuno et al. 1981 [10]	1	—	Hyperthyroidism	—	—	—	—

Pagliara et al. 1985 [11]	1	53/M	Hyperthyroidism and Addison's disease	Thyrotoxicosis	0.6	17.2 *μ*g/dL/417 ng/dL	Methimazole and cortisone

Daumerie et al. 1994 [12]	1	—	Nontoxic multinodular goiter	Euthyroid	—	—	—

Stanbury et al. 1954 [13]	2	—	1: hypothyroidism 1: nontoxic multinodular goiter Hypothyroidism	Myxedema	—	—	L-Thyroxine

Drucker et al. 1961 [14]	17	—	1: hypothyroidism	—	—	—	L-Thyroxine

Kuhl et al. 1961 [15]	4	—	Normal	Euthyroid	—	—	—

Brumlik and Maier 1972 [16]	1	39/F	Hypothyroidism	Myxedema	—	—	L-Thyroxine

Sagel et al. 1976 [17]	12	—	2: single thyroid nodules	Euthyroid	1.7	8.0 ± 1.6/25.8 ± 3.7	—

Lecomte et al. 1977 [18]	1	—	Normal	—	—	—	—

Henriksen et al. 1978 [19]	7	—	Normal	Euthyroid	Normal	Normal/normal	—

Tredici and Coletti 1978 [20]	2	—	Hypothyroidism	—	—	Low T4/—	L-Thyroxine

Okuno et al. 1979 [21]	1	53/F	Hyperthyroidism	—	—	—	Antithyroid drugs

Rioperez et al. 1979 [22]	2	—	1: hypothyroidism 1: nontoxic multinodular goiter	—	40	3.4–6.8 ng/100 mL/1.25–1.48 ng/mL	L-Thyroxine

Borda et al. 1982 [23]	1	—	Hyperthyroidism	—	—	—	—

Konagaya et al. 1983 [24]	1	—	Hyperthyroidism	—	—	—	—

Pizzi et al. 1985 [25]	12	—	1: hypothyroidism	—	Normal	Low T4/—	L-Thyroxine

Takase et al. 1987 [26]	26	—	Normal	—	Normal	Normal/normal	—

**Table 2:** Hyperparathyroidism associated with MD: review of the literature.

Authors	Number of cases	Age/sex	Hormonal status	Clinical features	Calcium mmol/L	Phosphate mmol/L	PTH pg/mL	Treatment
Ørngreen et al. 2012 [1]	97	25–65/4M-12F	16: hyperparathyroidism	Symptoms in 1 case	High: 2 cases 2.67 ± 0.03	Low: 7 cases 0.67 ± 0.02	High: 16 cases (16%)	Parathyroidectomy in 1 case

Molina et al. 1996 [2]	1	—	Hyperthyroidism and hyperparathyroidism	No symptoms	—	—	—	Parathyroid adenoma extirpation, subtotal thyroidectomy

Rosenberg et al. 1988 [3]	4	44-12-42-45/F	1: hyperparathyroidism (parathyroid hyperplasia) 1: hyperparathyroidism and neurofibromatosis 2: hyperparathyroidism and thyroid carcinoma	Weakness Hypercalcemia	High	—	High	Parathyroid adenoma extirpation

Passeri et al. 2013 [27]	44	44–56/M	8: hyperparathyroidism	2: hypercalcemia hypophosphatemia	2.37	0.74	High > 65 pg/mL	—

Harada et al. 1987 [28]	2	55–57/M-M	Hyperparathyroidism Parathyroid adenoma	No symptoms Hypercalcemia	High	—	High	Parathyroid adenoma extirpation

Kinoshita et al. 1997 [29]	24	36–58/6M-7F	13: pseudohypoparathyroidism	Hypocalcemia	2.05	1.005	1013.9	—

Garcia Delgado and Ruiz Galiana 1988 [30]	1	40/M	Hyperparathyroidism Parathyroid adenoma	Bone pain	—	—	—	Parathyroid adenoma extirpation

Middleton et al. 1989 [31]	1	52/F	Hyperparathyroidism Parathyroid hyperplasia	Symptomatic hypercalcemia	High: 4.05	0.35	5070	Parathyroidectomy

Downie and Jepson 1990 [32]	1	56/F	Hyperparathyroidism	No symptoms Hypercalcemia	High: 2.73	—	180	—

Bell et al. 1994 [33]	1	56/F	Hyperparathyroidism Parathyroid adenoma	Hypercalcemia	High	Low	High	Parathyroid adenoma extirpation
